# Bioinformatics Analysis of *LGR4* in Colon Adenocarcinoma as Potential Diagnostic Biomarker, Therapeutic Target and Promoting Immune Cell Infiltration

**DOI:** 10.3390/biom12081081

**Published:** 2022-08-06

**Authors:** Lijuan Wu, Xiaoxiao Tian, Hao Du, Xiaomin Liu, Haigang Wu

**Affiliations:** 1 Department of Gastroenterology, the First Affiliated Hospital, College of Clinical Medicine of Henan University of Science and Technology, Luoyang 471003, China; 2 Department of Orthopedic, the First Affiliated Hospital, College of Clinical Medicine of Henan University of Science and Technology, Luoyang 471003, China; 3 School of Life Sciences, Henan University, Kaifeng 475000, China

**Keywords:** LGR4, colon adenocarcinoma, bioinformatics analysis, tumorgenesis

## Abstract

Colon adenocarcinoma is one of the tumors with the highest mortality rate, and tumorigenesis or development of colon adenocarcinoma is the major reason leading to patient death. However, the molecular mechanism and biomarker to predict tumor progression are currently unclear. With the goal of understanding the molecular mechanism and tumor progression, we utilized the TCGA database to identify differentially expressed genes. After identifying the differentially expressed genes among colon adenocarcinoma tissues with different expression levels of *LGR4* and normal tissue, protein–protein interaction, gene ontology, pathway enrichment, gene set enrichment analysis, and immune cell infiltration analysis were conducted. Here, the top 10 hub genes, i.e., *ALB*, *F2*, *APOA2*, *CYP1A1*, *SPRR2B*, *APOA1*, *APOB*, *CYP3A4*, *SST*, and *GCG*, were identified, and relative correlation analysis was conducted. Kaplan–Meier analysis revealed that higher expression of *LGR4* correlates with overall survival of colon adenocarcinoma patients, although expression levels of *LGR4* in normal tissues are higher than in tumor tissues. Further functional analysis demonstrated that higher expression of *LGR4* in colon adenocarcinoma may be linked to up-regulate metabolism-related pathways, for example, the cholesterol biosynthesis pathway. These results were confirmed by gene set enrichment analysis. Immune cell infiltration analysis clearly showed that the infiltration percentage of T cells was significantly higher than other immune cells, and TIMER analysis revealed a positive correlation between T-cell infiltration and *LGR4* expression. Finally, COAD cancer cells, Caco-2, were employed to be incubated with squalene and 25-hydroxycholesterol-3-sulfate, and relative experimental results confirmed that the cholesterol biosynthesis pathway involved in modulating the proliferation of COAD tumorigenesis. Our investigation revealed that *LGR4* can be an emerging diagnostic and prognostic biomarker for colon adenocarcinoma by affecting metabolism-related pathways.

## 1. Introduction

Colon adenocarcinoma (COAD) is one of the major causes of cancer mortalities worldwide. COAD is the third most common tumor in men and the second most common tumor in women. Estimated by GLOBOCAN, 1,096,601 new diagnosed cases were identified, and COAD can lead to more than 550,000 deaths every year [1]. More clinical investigations showed that the 5-year survival rate of COAD patients is about 56% [2]. To further conduct analysis based on various stages of COAD, only 8–13% of patients bearing higher grades of COAD (stage III and IV) can survive more than 5 years [2]. Consequently, early diagnosis of COAD is critical for further providing clinical interventions, for example, chemotherapies or radiotherapies [3]. So far, many tumor-related genes are identified as prognostic biomarkers for early diagnosis, including *LAYN* [4], *HOXC11* [5], *CXCL11* [6], *CTHRC1* [7], and LINC00491 [8]. Thus, potentially prognostic molecular biomarkers are particularly required for improving COAD patient prognosis and providing more precise clinical interventions.

*LGR4*, also called *GPR48*, is another critical receptor for RANKL and involves modulating developmental pathways through canonical G-protein signaling [9,10]. To date, much literature reported that *LGR4* involves into prostate development and prostate stem cell differentiation, including male reproductive tract [11,12], female reproductive tract [13], eye [14], intestine [15], mammary gland [16], liver [17], epidermis [18], etc. In addition, *LGR4* also was identified as a critical modulator for regulating cell growth, cell death, cell fate, and motility [19,20]. Expression levels of *LGR4* are negatively correlated with p27 protein, which is a negative regulator of the E2F transcription factor [21,22,23]. Up-regulation of GPR48 (*LGR4*) can modulate phosphorylation levels of glycogen synthase kinase 3β through PI3K/Akt and MAPK/ERK1/2 pathway [24,25]. Therefore, the role of *LGR4* in COAD remains controversial and more investigation should be conducted to explore the potential molecular mechanism.

In this investigation, we explored the mRNA expression levels of *LGR4* in COAD patients from the TCGA database. To explore the impact of high expression *LGR4* on COAD, differentially expressed genes were identified and further bioinformatics analysis, for example, protein–protein interaction network analysis, gene ontology analysis, hub genes analysis, gene set enrichment analysis, and immune cell infiltration. Caco-2 cells were employed to be incubated with various concentrations of squalene and 25-hydroxycholesterol-3-sulfate. Cell viability and expression levels of downstream genes were examined by MTT assay and RT-PCR, respectively. Our investigation showed the role of overexpression of *LGR4* in the COAD tumorigenesis and immune cell infiltration, and it provides more evidence to support that *LGR4* may be a critical prognostic biomarker for early prognosis.

## 2. Materials and Methods

### 2.1. Data Acquisition

Genetic expression profiles and relatively clinical features of colon adenocarcinoma (*n* = 461) were obtained from the TCGA database (https://portal.gdc.cancer.gov/, Data Release 33.1—accessed on 31 May 2022), using R packages TCGAbiolinks and SummarizedExperiment. Detailed information on *LGR4* genetic mutation status and expression profiles compared to normal colon tissues were obtained from cBioPortal (https://www.cbioportal.org/, (accessed on 12 May 2022) and GEPIA 2.0 (http://gepia.cancer-pku.cn/, accessed on 3 December 2021) database. For the cBioPortal database, only TCGA (PanCan Atlas) database was employed for mutation information analysis (*n* = 594). For statistical analysis, the LIMMA method was employed to evaluate whether it can be considered a significant difference (*p* < 0.05)

### 2.2. Differentially Expressed Genes Screening and Protein Expression

The expression matrix of colon adenocarcinoma was divided into two individual groups: the low-expression group (*n* = 231) and the high-expression group (*n* = 230), in which the threshold value to identify as a high-expression group is set as median. Differentially expression genes (DEGs) were identified using R package (rio, dplyr and DESeq2 to identify the difference) with |log_2_FoldChange| ≥ 1.0 & padj < 0.05. Protein expression levels of *LGR4* in normal tissues and COAD tissues were obtained from the Human Protein Atlas (HPA, https://www.proteinatlas.org/, accessed on 11 November 2021).

### 2.3. Protein–Protein Interaction Network Analysis

Protein–protein interaction network analysis (PPI) was conducted on the STRING website (https://cn.string-db.org/, accessed on 30 December 2021). The up-regulated DEGs were submitted to the STRING website, and the minimum required interaction score was set as the highest confidence (*n* = 0.900) to exclude low confidence terms. The PPI network was further analyzed using Cytoscape 3.9.1 software (https://cytoscape.org/, Version 3.9.1, accessed on 30 December 2021), and hub genes were obtained using Cytoscape with plugin cytohubba. The Cytohubba is a plugin of Cytoscape to identify hub genes utilizing the PPI network. In this investigation, we used Maximal Clique Centrality (MCC) to identify the candidate hub gene of COAD.

### 2.4. Pathway Enrichment Analysis

To explore the key node pathways, we conducted pathway enrichment analysis using the Metascape website (https://metascape.org/gp/index.html#/main/step1, accessed on 18 December 2021). All the up-regulated DEGs were submitted to Metascape in the Homo sapiens database, and the top 20 ranked terms were analyzed. Gene ontology (GO) and pathway enrichment were further analyzed. For pathway enrichment analysis, the min overlap was set as 3, and the threshold value to consider as a significant difference is that the *p* value of pathway enrichment is less than 0.01. Other parameters were set as default.

### 2.5. Immune Cell Infiltration Analysis

To explore the immune cell infiltration levels, the expression matrix of COAD patients obtained from the TCGA database was divided into two groups by median value: low expression group and high expression. Estimation of immune cell infiltration was conducted using the ssGSEA method [26]. Correlation between *LGR4* expression level and immune cell infiltration level was conducted using the TIMER 2.0 database (http://timer.comp-genomics.org/, accessed on 12 May 2022) by the TIMER method. The correlation was conducted using Spearman’s method, in which positive correlation (*p* < 0.05, ρ > 0), negative correlation (*p* < 0.05, ρ < 0) and not significant (*p* > 0.05).

### 2.6. Gene Set Enrichment Analysis

To explore the impact of overexpression of *LGR4* in COAD, gene set enrichment analysis (GSEA) was conducted using GSEA software (http://www.gsea-msigdb.org/gsea/index.jsp, accessed on 22 March 2022) and the GSVE package. The threshold value of GSEA analysis considered as significance was FDR < 0.25 and NOM *p* value < 0.05 and |Normalized enrichment score, NES| ≥ 1.0.

### 2.7. Cell Culture

Caco-2 cell lines were purchased from the cell bank of the Chinese Academy of Sciences. Cells were cultured in high glucose DMEM medium (Hyclone, SH30022.01) containing 10% FBS (fetal bovine serum, Gibco, 10099-141), 1% penicillin/streptomycin (Gibco, C14-15070-063) in 5% CO_2_ at 37 °C. Squalene (J&K, Shanghai, China) and 25-hydroxycholesterol-3-sulfate (TargetMol, Shangahi, China) were suspended in DMEM, and then Caco-2 cells were incubated with drugs following the given concentration. Cell viability of Caco-2 post treatment was examined using MTT (Thiazolyl blue tetrazolium bromide, Cat. IM0280, Solarbio Life Science, Beijing, China) method following the manufacture manual.

### 2.8. RT-PCR Analysis

RNA samples of cells were collected by using TreliefTM RNAprep FastPure Tissue & Cell Kit purchased from TSINGKE Biological Technology. RNA samples were reverse transcribed into cDNA using PrimeScriptTM RT Master Mix (Perfect Real Time) from TaKaRa. cDNA samples were amplified and calculated by a LightCycler 480 II (Roche, Basel, Switzerland) system. The reaction was performed by the following process: 95 °C for 2 min, followed by 45 cycles at 95 °C for 15 s and 62 °C for 1 min. The sequence of interested gene primers is listed in Table 1. All the Ct values were controlled to lower than 40, indicating the efficiency of gene amplification. All the RT-qPCR assays obeyed the MIQE guidelines [27].

### 2.9. Statistical Methods

All statistical analyses were conducted using the R package unless otherwise mentioned. Data are presented as mean ± S.D. Threshold value considered as significance is *p* < 0.05 or FDR *p* value < 0.05.

## 3. Results

### 3.1. Clinical Characteristics and LGR4 Expression of COAD Patients

**Figure 1:**
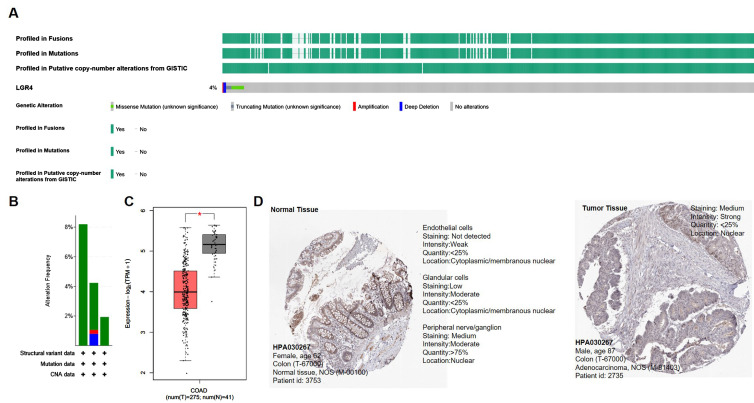
Overexpression of *LGR4* in COAD tumor compared to normal tissue. (**A**) Genetic status of *LGR4* in COAD patients obtained from the TCGA database. (**B**) Cancer types depended on gene alteration frequency obtained from TCGA database. (**C**) Expression levels of *LGR4* genes in COAD patients were compared to normal colon tissue, and difference was conducted using LIMMA method (*, *p* < 0.05). (**D**) Expression profiles of *LGR4* protein in normal colon tissue and COAD tissue were obtained from HPA database.

As shown in Table 2, the average age of newly diagnosed COAD patients is 66.92 ± 13.08, indicating that COAD patients are older than other tumor-bearing patients. Other clinical characteristics of COAD patients are presented in Table 2. Among these COAD patients, the mutation frequency of the *LGR4* gene is only 4% (Figure 1A,B). This mutation frequency is far less than other critical oncogenes, for example, *KRAS* [28,29] or *TP53* [30]. The low mutating frequency of the *LGR4* gene in the COAD patients indicated that gene expression level may be associated with modulating the tumorigenesis process.

Subsequently, we examined whether the expression levels of *LGR4* mRNA in the COAD patients are changed compared to normal colon tissue (GTEx database). As given in Figure 1C, we can find that the expression level of *LGR4* in the COAD patients was significantly lower than in normal tissues (*p* < 0.05). Finally, protein expression of LGR4 in the tissue samples of COAD was analyzed using the HPA database, as given in Figure 1D. Compared to normal tissue (Figure 1D, left panel), LGR4-positive regions in the COAD tumor tissues by immunohistochemistry (IHC) method are significantly lower than normal colon tissues, consistent with mRNA result. In contrast to oncogenes with higher expression levels, lower expression of *LGR4* in COAD may be led by several reasons, for example, inhibition of transcription factor activity and mutation of up-stream genes. To test the correlation between low expression of LGR4 and COAD tumorigenesis, more investigation should be conducted.

### 3.2. High Expression of LGR4 in COAD Worsens the Overall Survival of Patients

**Figure 2:**
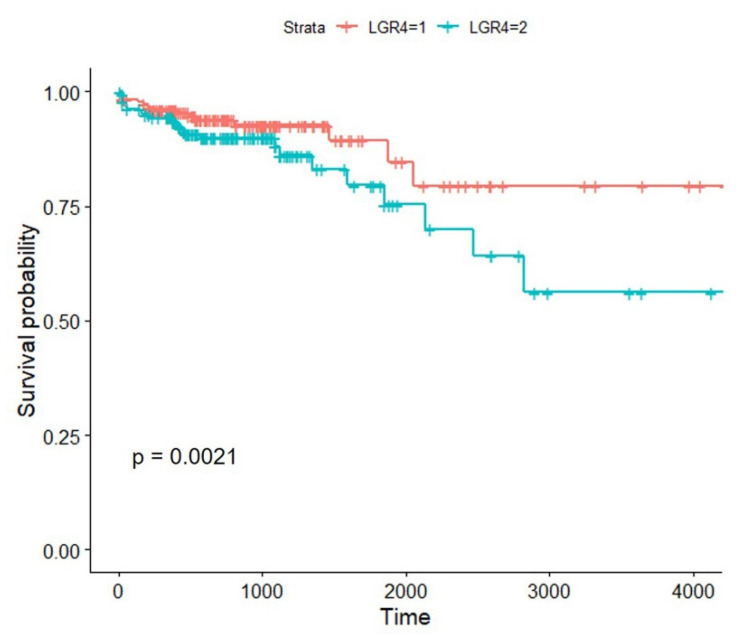
Kaplan–Meier analysis of COAD patients depended on *LGR4* expression. Cut-off value is median value of *LGR4* mRNA expression level. LGR4–1 is attributed to low expression of *LGR4* in the COAD patients, and LGR4–2 is attributed to high expression of *LGR4* in COAD patients. The curve comparison with the log-rank (Mantel-Cox) test revealed statistically significant differences as shown on graph (*p* = 0.0021).

To investigate how *LGR4* affects the survival of COAD patients, we conducted the Kaplan–Meier analysis to utilize survival data obtained from the TCGA database. The cut-off value to divide COAD patients into two groups is median. As given in Figure 2, the survival of COAD patients with higher expression of *LGR4* is significantly worse than low expression group (*p* = 0.0021), indicating that higher expression of *LGR4* in the COAD is associated with overall survival of COAD patients.

### 3.3. Identification of Differentially Expressed Genes

**Figure 3:**
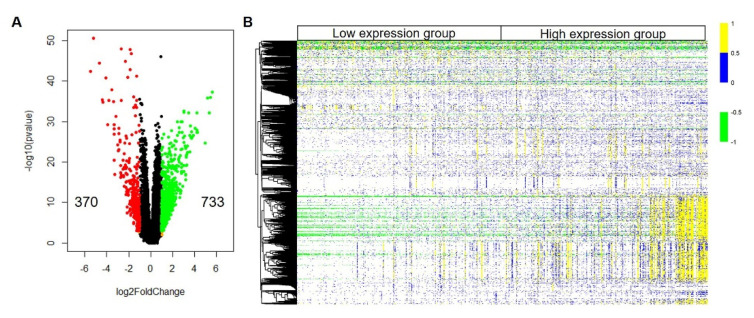
Expression profile of identified genes in *LGR4*-low expression group and *LGR4*-high expression. (**A**) Volcano plot of genes in *LGR4*-high expression group compared to *LGR4*-low expression group. Threshold value of differentially expressed gene is |log_2_FoldChange| > 1.0 & *p*-value < 0.001. (**B**) Heatmap of differentially expressed genes identified in *LGR4*-high expression group compared LGR4-low expression group.

To explore how the higher expression of *LGR4* involves affecting the overall survival of COAD patients, differentially expressed genes (DEGs) between high and low expression groups were first identified. The method to identify DEGs is DESeq2 software in Rstudio. As given in Figure 3A, we can observe that there are 1103 DEGs compared to low expression group (threshold value to identify DEGs is |log_2_FoldChange| ≥ 1.0 & *p*-adj < 0.05). In total, 370 DEGs and 733 DEGs were attributed as down-regulation and up-regulation, respectively. Heatmap of DEGs expression levels is presented in Figure 3B. After clustering analysis, we can find that these DEGs can be divided into several groups (Figure 3B). DEGs in the same cluster implied that these genes may be a higher correlation with each other. Consequently, it is necessary to explore biological functions of these DEGs, which may provide more information about the involvement of *LGR4* in COAD tumorigenesis.

### 3.4. Protein–Protein Interaction Network Analysis

**Figure 4:**
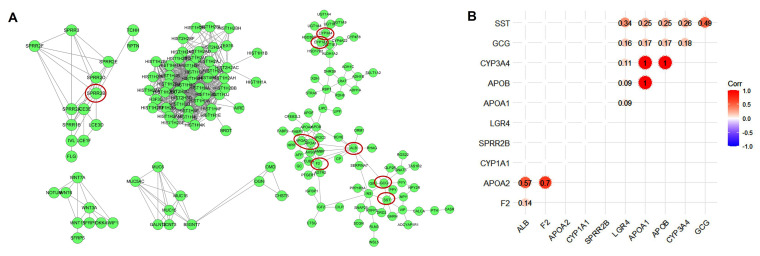
(**A**) Protein–protein interaction network of DEGs identified in *LGR4*-high expression group compared to *LGR4*-low expression group. The red circle-labeled hub genes of DEGs are *ALB*, *F2*, *APOA2*, *CYP1A1*, *SPRR2B*, *APOA1*, *APOB*, *CYP3A4*, *SST*, and *GCG*. (**B**) Heatmap of Pearson’s correlation matrix of hub genes in *LGR4*-high expression group compared to *LGR4*-low expression group. Threshold value identified as significant is *p* value < 0.05.

Up-regulated and down-regulated DEGs can provide more precise and useful information about how LGR4 is involved in affecting molecular networks in COAD. However, as given in Figure 3B, up-regulated DEGs may display a higher correlation to regulating molecular network compared to down-regulated DEG. To explore the interaction networks of these up-regulated DEGs, we conducted protein–protein network interaction (PPI) analysis. As given in Figure 4A, PPI network can be attributed to five groups, implying a higher correlation between these up-regulated DEGs. Then, we identified nine hub genes, i.e., *ALB*, *F2*, *APOA2*, *CYP1A1*, *SPRR2B*, *APOA1*, *APOB*, *CYP3A4*, *SST*, and *GCG*. Among these hub genes, we further analyzed the correlation compared to *LGR4* expression. As given in Figure 4B, we can find that only several hub genes, i.e., *APOP1*, *APOB*, *CYP3A4*, *GCG*, and *SST*, displayed a positive correlation with *LGR4*. *SST*, which encoded somatostatin, is an important hormone to maintain homeostasis of intestine function, and relative analogs also can be used as therapeutic agents for gastrointestinal angiodysplasias. *APOP1*, *APOB*, and *GCG* are highly associated with nutrient adsorption and metabolism processes. These results implied that higher expression of *LGR4* mRNA in COAD may activate a local immune response.

### 3.5. Modulation of Pathways by Up-Regulated LGR4 in COAD

**Figure 5:**
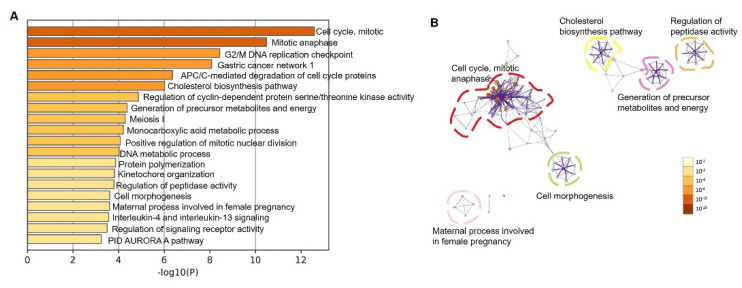
(**A**) Bar graph of enriched terms across up-regulated DEGs, colored by *p*-values. (**B**) Network of enriched terms colored by *p*-value, where terms containing more genes tend to have a more significant *p*-value.

To further analyze the impact of *LGR4* on molecular networks, we conducted gene ontology (GO) analysis using up-regulated DEGs using the Metascape database. As given in Figure 5A, the top 10 ranked GO terms can be attributed as cell cycle (mitotic), mitotic anaphase, G2/M DNA replication checkpoint, gastric cancer network 1, APC/C-mediated degradation of cell cycle proteins, cholesterol biosynthesis pathway, regulation of cyclin-dependent protein serine/threonine kinase activity, generation of precursor metabolites and energy, meiosis I, and monocarboxylic acid metabolic process. Most of these terms are associated with cell-cycle-related annotations, implying that these up-regulated DEGs may involve cell proliferation processes. To further explore the correlation among these pathways, we also conducted pathway enrichment analysis, as given in Figure 5B. Most terms are attributed to cell cycle and mitotic anaphase clusters. Other key nodes enriched were the cholesterol biosynthesis pathway and generation of precursor metabolites and energy, presented by yellow and purple circles in Figure 5B. These results implied that overexpression of *LGR4* in the COAD pathway may affect the cholesterol biosynthesis pathway and generation of precursor metabolites and energy, and it may further modulate the downstream signaling pathways.

**Figure 6:**
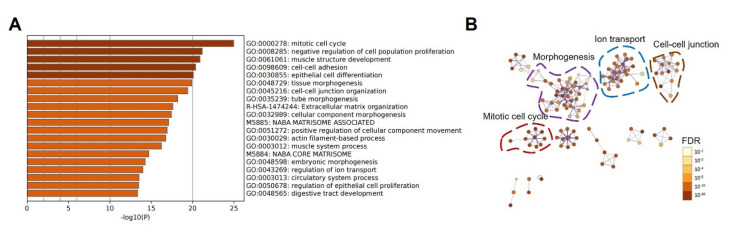
(**A**) Bar graph of enriched terms across up-regulated DEGs between COAD and normal tissues, colored by *p*-values. (**B**) Network of enriched terms using DEGs between COAD and normal tissues, where terms containing more genes tend to have a more significant *p*-value.

To evaluate whether the cholesterol biosynthesis pathway is associated with promoting tumorigenesis, we further explored bioinformatics analysis of DEGs identified between COAD and normal tissue. Firstly, 1558 up-regulated DEGs were identified, and GO analysis was conducted (Figure 6A). As given in Figure 6A, metabolism-related pathways were not identified in top−20 enriched pathways. Among these pathways, most of these annotations can be attributed to cell-cycle-related and tissue development-related pathways. To further validate our hypothesis, we conducted pathway enrichment as given in Figure 6B. Clustering results clearly showed that major clusters are mitotic cell cycle, morphogenesis, ion transport, and cell–cell junction, respectively. These results highly confirmed our hypothesis that metabolism-related pathways were associated with worsening overall survival of COAD patients caused by a higher expression level of LGR4.

### 3.6. Gene Set Enrichment Analysis

To identify which pathway was associated with COAD tumorigenesis, we conducted gene set enrichment analysis (GSEA) to identify up-regulated pathway. Here, we utilized two gene sets for GSEA analysis, which are gene ontology (GO) and Kyoto Encyclopedia of Genes and Genomes (KEGG), to perform GSEA.

**Figure 7:**
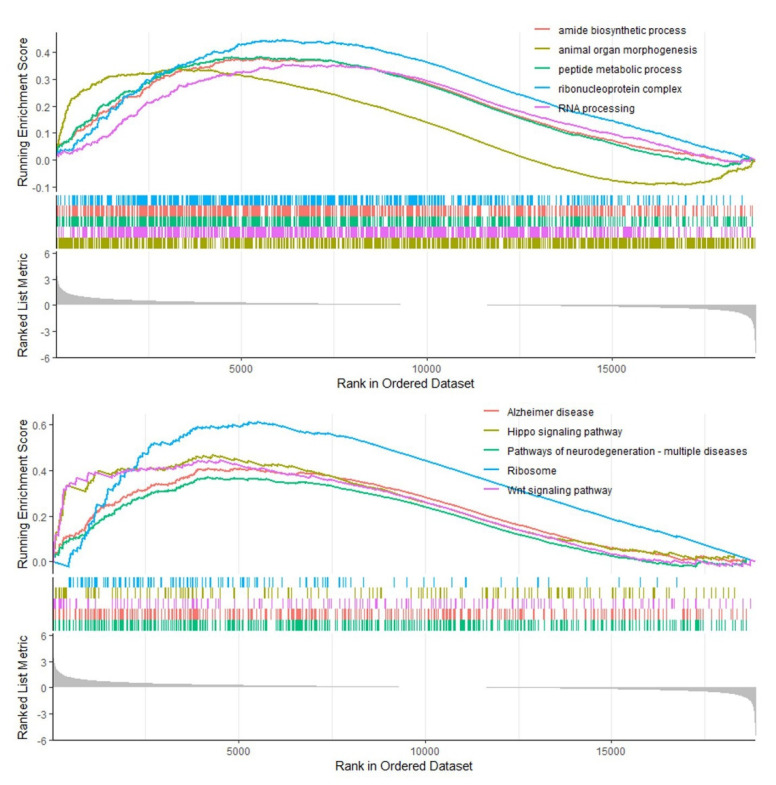
GSEA analysis of COAD patient in correlation with up-regulated pathways using GO and KEGG gene set. For the analysis, all genes were ranked by Pearson’s correlation to controls. Threshold value of GSEA annotation identified as significant difference is |normalized enrichment score (NES)| > 1.0 & FDR *p*-value < 0.05.

As given in the upper panel of Figure 7, the top 5 enriched GO terms ranked by normalized enrichment score (NES) were presented. These terms are amide biosynthetic process, animal organ morphogenesis, peptide metabolic process, ribonucleoprotein complex, and RNA process. Amide biosynthetic process and peptide metabolic process can be attributed to metabolic process, implying that up-regulation of *LGR4* in COAD may promote the protein synthesis process. Ribonucleoprotein complex and RNA processing can be attributed to the transcription process, demonstrating that up-regulation of *LGR4* may promote gene expression levels of oncogenes in COAD patients. These results implied that up-regulation of *LGR4* may participate in modulating tumor-related gene expression and metabolism-related processes.

As mentioned above, the top 5 enriched KEGG pathways were presented in the down panel of Figure 7, i.e., Alzheimer’s disease, Hippo signaling pathway, pathways of neurodegeneration-multiple disease, Ribosome, and Wnt signaling pathway, respectively. To our knowledge, Alzheimer’s disease and pathways of neurodegeneration-multiple disease are highly associated with inflammation response, indicating the critical role of inflammation response in the highly *LGR4*-expressed COAD. The Hippo signaling pathway, which is a critical pathway for the modulation of organ size [31], can be substantially considered as up-regulation, demonstrating that *LGR4* may activate this pathway to modulate the proliferation of tumor size by Hippo signaling pathway. Owing that *LGR4* is natural receptor of Wnt [32], overexpression of *LGR4* also can activate the Wnt signaling pathway, which is consistent with GSEA analysis results.

### 3.7. Immune Cell Infiltration Analysis

**Figure 8:**
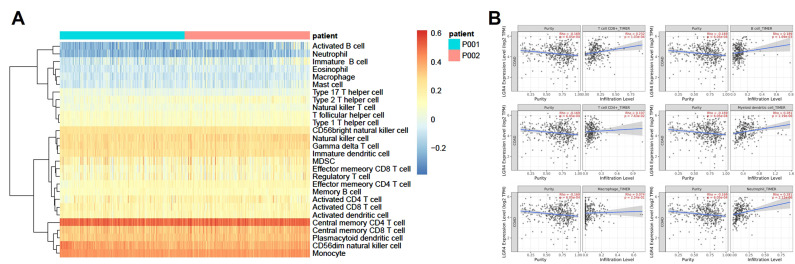
(**A**) Unsupervised clustering analysis of COAD based on ssGSEA score of 24 immune cell types. P001 is attributed to COAD patients with lower expression level of *LGR4*, and P002 is attributed to COAD patients with higher expression level of *LGR4*. (**B**). Correlation between *LGR4* expression level and infiltration level of major immune cells.

Therapeutic treatment targeting the tumor microenvironment can significantly improve clinical outcomes [33]. Recent investigation strongly suggested that the infiltration levels of immune cells into tumor tissues may be regulated by intrinsic pathways and relative regulatory networks. Here, we examined the degrees of immune cell infiltration from the TCGA database, and ssGSEA was employed to evaluate immune cell infiltration. As given in Figure 8A, ssGSEA results indicated that the relative degrees of immune infiltration were not significantly affected by expression levels of *LGR4* in COAD. However, we also found that infiltration degrees of T cells in total COAD patients were significantly higher than in other types of immune cells. Then, we employed the TIMER2.0 database to estimate the correlation between major immune infiltration and *LGR4* expression, as shown in Figure 8B. Analysis results clearly showed that T cell CD8^+^ (Rho = 0.232, *p* = 1.03 × 10^−4^), B cell (Rho = 0.189, *p* = 1.69 × 10^−3^), myeloid dendritic cell (Rho = 0.281, *p* = 2.19 × 10^−6^), and neutrophil (Rho = 0.281, *p* = 2.12 × 10^−6^) displayed a positively correlation with LGR4 expression in COAD.

### 3.8. Analysis of Cholesterol Biosynthesis in COAD

**Figure 9:**
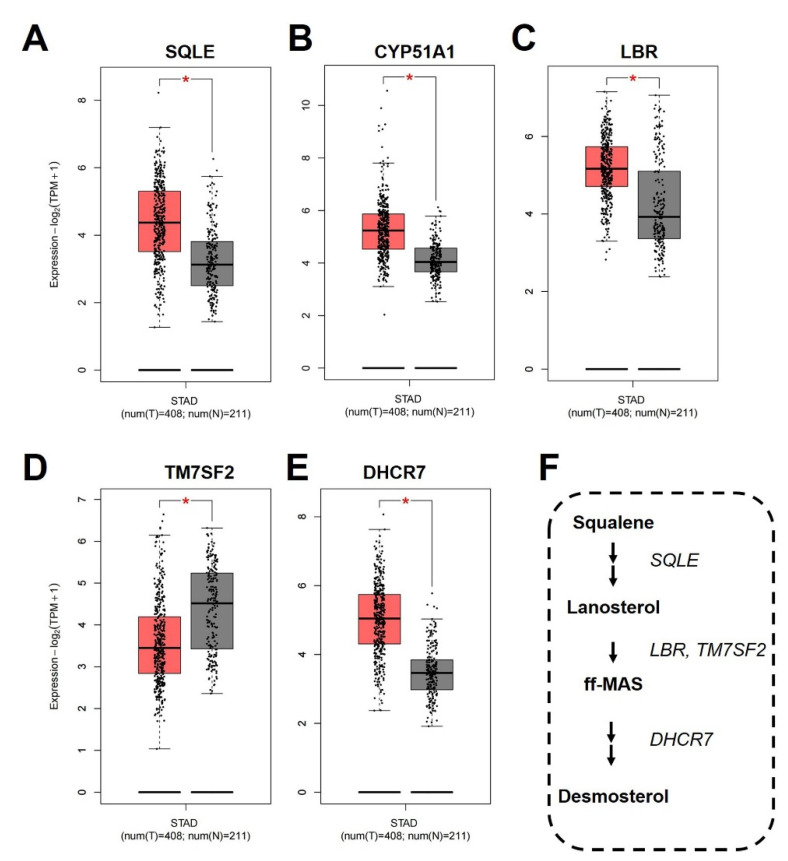
Expression levels of differentially expressed genes in cholesterol biosynthesis pathway: (**A**) *SQLE*; (**B**) *CYP51A1*; (**C**) *LBR*; (**D**) *TM7SF2*; (**E**) *DHCR7*. (**F**) Schematic illustration of the Bloc and Kandutsch–Russell cholesterol biosynthesis pathway for the enzymatic conversion of squalene to desmosterol. Statistical method to consider as significant difference is LIMMA with *, *p* < 0.05.

As above-mentioned, cholesterol biosynthesis pathway may be associated with modulating tumorigenesis of COAD. Subsequently, we analyzed expression levels of catalytic enzymes in the cholesterol biosynthesis pathway, which contained 12 critical enzymes [34]. As given in Figure 9A,E, expression levels of only five genes, i.e., *SQLE*, *CYP51A*, *LBR*, *TM7SF2*, and *DHCR7*, in the COAD tissues presented a significant difference compared to normal tissue using the GEPIA2.0 database. The roles of these enzymes in cholesterol biosynthesis are different (Figure 9F), and expression levels of four genes (*SQLE*, *CYP51A*, *LBR*, and *TM7SF2*) were significantly higher than in normal tissues. Higher expression of these enzymes indicated that synthesis of lanosterol and ff-MAS is much higher than in normal tissues. However, expression levels of *DHCR7* in tumor tissues were significantly lower than in normal tissues, implying that components of desmosterol in tumors may be lower than in normal tissues.

**Figure 10:**
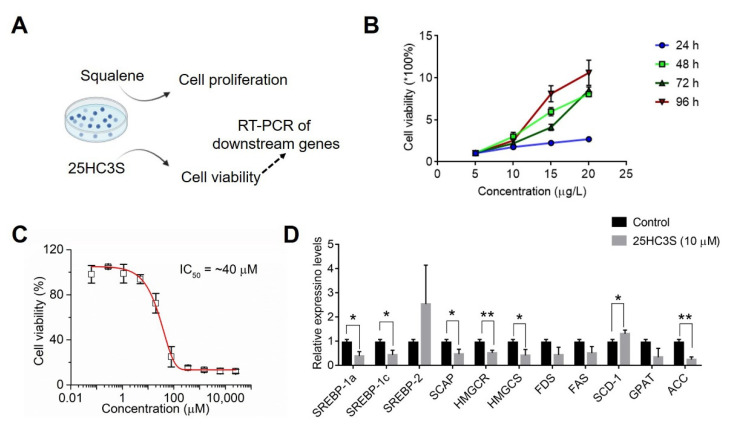
Effect of squalene and 25-hydroxycholesterol-3-sulfate (25HC3S) on Caco-2 cells. (**A**) Schematic illustration for effects of cholesterol precursor and inhibitors on cancer cells. (**B**) Cell viability of cancer cells treated with different concentrations of squalene at different times (24 h, 48 h, 72 h, and 96 h). (**C**) Cell viability of cancer cells treated with different concentrations of 25HC3S at 48 h. (**D**) Expression levels of targeted genes of cholesterol biosynthesis pathway. Data are presented as Mean ± Standard Error (three biological replicates per group). Statistical significance by Student’s *t* test; *, *p* < 0.05; **, *p* < 0.01.

To validate the role of cholesterol biosynthesis in COAD, we further used squalene and 25HC3S, which are respective precursor and inhibitor of cholesterol biosynthesis, to treat COAD cancer cell line Caco-2 cells (Figure 10A). As shown in Figure 10B, we can observe that different concentrations of squalene can promote the time-course proliferation of Caco-2. Then, Caco-2 cells were incubated with various concentrations of 25HC3S at 48 h, and the IC_50_ value of 25HC3S on Caco-2 cells was about 40 μM. After incubation with 10 μM 25HC3S for 48 h, expression levels of downstream genes, as shown in Figure 10D, were validated by RT-PCR. Except for *SREBP-2* and *SCD-1*, most of the downstream genes were significantly down-regulated, demonstrating that blockade of cholesterol biosynthesis pathway can inhibit oncogene expression, which may be employed for anti-proliferation and COAD therapy.

## 4. Discussion

COAD is a particularly difficult cancer with a high mortality rate in advanced stages [35,36,37]. Here, we conducted the integral bioinformatic analysis to identify a new biomarker for COAD early diagnosis and prognosis. Although this biomarker in COAD is lowly expressed compared to normal tissue, a higher expression level of LGR4 is associated with lower survival of COAD patients. By conducting PPI analysis, GSEA, and pathway enrichment, we observed that the metabolism pathway, i.e., cholesterol biosynthesis pathway, may be involved in tumor progression, confirmed by in vitro assays.

Firstly, we explored *LGR4* expression status in COAD patients (Figure 1) in the TCGA database, and statistical analysis clearly showed that mutation of the *LGR4* gene in COAD was very low (~4%). Compared to normal colorectal tissues, expression levels of *LGR4* are significantly lower in tumor tissues. In contrast to other oncogenes, this low-expression *LGR4* is determined by biological function and downstream modulators, i.e., the Wnt/β-catenin pathway and cholesterol biosynthesis pathway. To conduct K–M analysis, the survival curve clearly showed that higher expression of *LGR4* is associated with lower survival of COAD patients compared to lower expression of *LGR4*. This result is very interesting, and it may imply that higher expression of *LGR4* may up-regulate several pathways to activate proliferation to worsen COAD patients’ survival.

Then, we firstly identified the DEGs between the higher expression group and the lower expression group. There were 370 down-regulated DEGs and 733 up-regulated DEGs, respectively. To conduct PPI analysis using up-regulated DEGs, we identified 10 hub genes, i.e., *ALB*, *F2*, *APOA2*, *CYP1A1*, *SPRR2B*, *APOA1*, *APOB*, *CYP3A4*, *SST*, and *GCG*. Among these hub genes, *SST*, *GCG*, *APOA1*, and *APOB* can be attributed to metabolism processes, implying that overexpression of *LGR4* may involve modulating metabolic processes.

Furthermore, we determined the critical role of *LGR4* in modulating relative pathways using pathway enrichment and GSEA analysis. As given in Figure 5 and Figure 6, cell-cycle-related pathways were significantly enriched as major pathway clusters. Moreover, the cholesterol biosynthesis pathway and relative metabolism pathway were enriched, which is consistent with GSEA results (Figure 7) and confirms the likely involvement of *LGR4* in modulating metabolism-related pathways.

To our knowledge, metabolism-related pathways are highly associated with the tumor microenvironment [38,39,40]. We further explored the association between immune cell infiltration and *LGR4* expression. Using ssGSEA analysis, we can observe that most T cells in bulk COAD tissues were significantly enriched compared to B cells, implying that higher infiltration levels of T cells may involve in COAD tumorigenesis. Consequently, we conducted immune cell infiltration analysis using the ssGSEA and TIMER database. As given in Figure 8B, correlation results clearly showed that CD8^+^ T cells, CD4^+^ T cells, myeloid dendritic cells, and neutrophil cells displayed a positive correlation with *LGR4* expression, confirming that higher expression of *LGR4* can promote the infiltration degrees of these immune cells. Interestingly, B cells also displayed a positive correlation with *LGR4* expression.

To validate the hypothesis that the cholesterol biosynthesis pathway involves modulating COAD tumorigenesis, squalene as initiating precursor for cholesterol synthesis was employed to treat Caco-2 cells. Cell viability (Figure 10B) clearly showed that over-loading of squalene can promote cellular proliferation of Caco-2 cells for different incubation times. Then, an inhibitor of cholesterol biosynthesis pathway 25HC3S was employed to treat Caco-2 cells, and cell viability of Caco-2 (Figure 10C) displayed the dosage-dependent anti-proliferation. To further examine the effects of 25HC3S on Caco-2 cells, RT-PCR was employed to measure the downstream genes, which confirmed that the SREPB-mediated pathway was significantly inhibited. SREPB is modulated by the cholesterol biosynthesis pathway [41]. These results implied that the LGR4-modulating cholesterol biosynthesis pathway can be a potential therapeutic target for COAD therapy.

## 5. Conclusions

In our investigation, we firstly found that expression levels of *LGR4* in COAD tissues are significantly lower than in adjacent normal tissues, confirming that loss function of *LGR4* may be associated with promoting tumorigenesis. Then, K–M analysis obtained from TCGA clearly showed that higher expression of *LGR4* in COAD patients can significantly worsen the overall survival. Further investigation demonstrated that higher expression of *LGR4* can promote cholesterol biosynthesis-related and cell-cycle-related pathways, implying that *LGR4* may involve in regulating tumorigenesis by affecting metabolism-related pathways. Cell viability and expression levels of downstream genes in Caco-2 cells incubated with squalene and 25HC3S revealed that cholesterol biosynthesis is involved in COAD tumorigenesis. Our results suggested therapeutic, diagnostic, and prognostic potentials of *LGR4* for COAD therapy.

## Figures and Tables

**Table 1:** Primer sequence of selected genes investigated in this paper.

Name	Forward Sequence	Reverse Sequence
*GAPDH*	CAATGACCCCTTCATTGACC	TTGATTTTGGAGGGATCTCG
*HMGR*	GTCATTCCAGCCAAGGTTGT	GGGACCACTTGCTTCCATTA
*HMGCS*	CAGCTGCTGTVTTVAATGCTGTTA	AGCTACTGCTCCAACTCGGCATC
*FAS*	TGTGGACATGGTCACGGAC	GGCATCAAACCTAGACAGGTC
*ACC1*	TCGCTTTGGGGGAAATAAAGTG	ACCACCTACGGATAGACCGC
*SREBP-1a*	GCGCCATGGAGGAGCTGCCCTTCG	GTCACTGTCTTGGTTGTTGATG
*SREBP-1c*	TGCGGACGCAGTCTGGGCAAC-3	GTCACTGTCTTGGTTGTTGATG
*SREBP-2*	AACGGTCATTCACCCAGGTC	GGCTGAAGAATAGGAGTTGCC
*GPAT*	ATCTTCAGAACAGCAAAATCGAAA	CAGCGGAAAACTCCAAATCC
*SCD1*	TTCTTGCGATACACTCTGGTGC	CGGGATTGAATCTTCTTGTCGT
*FDS*	TCCATGGCGGATCTGAAGTCAACT	CATCCAGTCTTTGTCCATGTATCTG
*SCAP*	ACTGGGCATCATCCTCATTG	GGCACTGTCTGGTTCTCTGG

**Table 2:** Clinical features of COAD in TCGA database.

Clinical Factor	TCGA Database
Age (years, mean ± SD)	66.92 ± 13.08
Sex (female, male)	230/285
Stage of COAD	
Stage I/IA	75/1
Stage II/IIA/IIB/IIC	30/137/10/1
Stage III/IIIA/IIIB/IIIC	20/8/60/41
Stage IV/IVA/IVB	46/17/2
NA	14
Tissue_or_organ_of_origin	
Ascending colon	91
Cecum	88
Colon, NOS	104
Descending colon	17
Hepatic flexure of colon	12
Rectosigmoid junction	7
Sigmoid colon	114
Splenic flexure of colon	5
Transverse colon	21
NA	2
Primary_diagnosis	
Adenocarcinoma with mixed subtypes	1
Adenocarcinoma with neuroendocrine differentiation	1
Adenocarcinoma, NOS	388
Adenosquamous carcinoma	1
Carcinoma, NOS	1
Mucinous adenocarcinoma/	65
Papillary adenocarcinoma, NOS	2
NA	2
Death (alive/death/no report)	357/102/2
Radiation therapy (yes/no/no report)	379/12/70
Pharmaceutical therapy (yes/no/no report)	150/241/70

Note: SD—standard deviation; NA—not available.

## Data Availability

The datasets used and/or analyzed during the current study are available from the corresponding author on reasonable request. Images produced by Rstudio software and codes for R script have been uploaded to GitHub: https://github.com/johnwu0536/Biomolecules_codes, to date 1 August 2022.

## Abbreviations

- *COAD*: *Colon Adenocarcinoma*
- TCGA: The Cancer Genome Atlas
- TIMER: Tumor Immune Estimation Resource
- RANKL: Receptor activator of nuclear factor kappa-B ligand
- DEGs: Differentially expressed genes
- PPI: Protein–protein interaction
- GO: Gene Ontology
- GSEA: Gene Set Enrichment Analysis
- ssGSEA: Single-sample GSEA
- DMEM: *Dulbecco’s Modified Eagle Medium*
- MTT: 2,5-diphenyl-2H-tetrazolium bromide
- GTEx: The Genotype-Tissue Expression
- KEGG: Kyoto Encyclopedia of Genes and Genomes
- NES: Normalized enrichment score
- ff-MAS: Follicular fluid meiosis-activating sterol
- 25HC3S: 25-hydroxycholesterol-3-sulfate
- HPA: The Human Protein Atlas
- K–M analysis: Kaplan–Meier analysis
- FDR: False Discovery Rate
